# Human-Centered Design of Mobile Health Apps for Older Adults: Systematic Review and Narrative Synthesis

**DOI:** 10.2196/29512

**Published:** 2022-01-14

**Authors:** Zethapong Nimmanterdwong, Suchaya Boonviriya, Pisit Tangkijvanich

**Affiliations:** 1 Department of Biochemistry Faculty of Medicine Chulalongkorn University Bangkok Thailand; 2 Center of Excellence in Hepatitis and Liver Cancer Faculty of Medicine Chulalongkorn University Bangkok Thailand

**Keywords:** connected health, development, elderly, HCD, mHealth, older adults, review, telehealth, UCD, usability, design, human-centered, app

## Abstract

**Background:**

The world is aging. The number of older patients is on the rise, and along with it comes the burden of noncommunicable diseases, both clinical and economic. Attempts with mobile health (mHealth) have been made to remedy the situation with promising outcomes. Researchers have adopted human-centered design (HCD) in mHealth creation to ensure those promises become a reality.

**Objective:**

This systematic review aims to explore existing literature on relevant primary research and case studies to (1) illustrate how HCD can be used to create mHealth solutions for older adults and (2) summarize the overall process with recommendations specific to the older population.

**Methods:**

We conducted a systematic review to address the study objectives. IEEE Xplore, Medline via Ovid, PubMed, and Scopus were searched for HCD research of mHealth solutions for older adults. Two independent reviewers then included the papers if they (1) were written in English, (2) included participants equal to or older than 60 years old, (3) were primary research, and (4) reported about mHealth apps and their HCD developments from start to finish. The 2 reviewers continued to assess the included studies’ qualities using the Mixed Methods Appraisal Tool (MMAT). A narrative synthesis was then carried out and completed.

**Results:**

Eight studies passed the eligibility criteria: 5 were mixed methods studies and 3 were case studies. Some studies were about the same mHealth projects with a total of 5 mHealth apps. The included studies differed in HCD goals, target groups, and details of their HCD methodologies. The HCD process was explored through narrative synthesis in 4 steps according to the International Standardization Organization (ISO) standard 9241-210: (1) understand and specify the context of use, (2) specify the user requirements, (3) produce design solutions to meet these requirements, and (4) evaluate the designs against requirements. The overall process and recommendations unique to older adults are summarized logically with structural order and time order based on the Minto pyramid principle and ISO 9241-210.

**Conclusions:**

Findings show that HCD can be used to create mHealth solutions for older adults with positive outcomes. This review has also summarized practical HCD steps and additional suggestions based on existing literature in the subfield. However, evidence-based results are still limited because most included studies lacked details about their sampling methods and did not set objective and quantifiable goals, leading to failure to draw significant conclusions. More studies of HCD application on mHealth for older adults with measurable design goals and rigorous research strategy are warranted.

## Introduction

### Background

The word “mHealth,” or “mobile health,” has been rising in popularity. A search of the term in an academic research database bears tens of thousands of results in 2020 alone. It is being studied as a medical intervention for arthritis [1], asthma [2], cancer [3], cardiovascular diseases [4], chronic kidney diseases [5], diabetes [6], multiple sclerosis [7], and various psychiatric diseases [8]. The idea of health care through mobile technology indeed accounts for its reputation. The World Health Organization defines mHealth as the “medical and public health practice supported by mobile devices, such as mobile phones, patient monitoring devices, personal digital assistants, and other wireless devices” [9]. mHealth is often brought up together with its broader term *telehealth* or *telemedicine*, which essentially means the practice of any kind of medicine with the help of technology across the distance [10]. With the COVID-19 pandemic, where social distancing is key, such digitalization of health care is becoming more relevant than ever [11].

mHealth and telehealth are the means to achieve timely and accurate health management; they help enable a seamless sharing of medical information between all those involved, creating the so-called connected health environment that the current trend strives for [12]. Successful integration of such innovations is believed to ensure universal health coverage, reduce health care costs, and improve clinical outcomes [9]. There were 5.2 billion mobile phone users at the end of 2019 with the estimation that the number will reach 5.8 billion by 2025, roughly 70% of the entire human population [13]. Diffusion of health care through a mobile medium in such a large populace will surely guarantee impact on a global scale. Real-world mHealth implementations across the globe are committed to educating patients, offering easier access to medical care, improving medical data storage and transfer, empowering health care providers, and boosting the efficiency of its institutions [14]. The synthesis of clinical evidence in the field is also on the rise. A meta-analysis of 11 lifestyle modification apps reported a significant reduction in the mean HbA_1c_ of the users in both short- and long-term observations [15]. Self-management interventions in 24 studies were shown to be able to decrease both systolic and diastolic blood pressures in patients with hypertension [16]. One systemic review that focused on pediatric asthma management reported increased treatment adherence in 13 studies, reduced exacerbations in 5, and improved quality of life in 4 [17].

Although mHealth has remarkable potential, most projects cannot scale to their own target population and fail to achieve the intended results. This can be attributed to (1) poor understanding of the end users and (2) failing business models [18]. Barriers to user adoption of mHealth can range from an individual level to a higher level of the policy governing its use. However, while policy barriers tend to impede new innovations or hinder the successful ones from a larger adoption [19], user-related barriers pose a more tangible challenge as no one might use the technology in the first place. A survey in the United States showed that about half of those who have downloaded health apps stop using them eventually [20]. The cause of this begins when inadequate user involvement makes it impossible to draft concise software requirements [21], which results in poor user acceptance and failure to scale [22].

These issues get even more complicated with older adults. The United Nations defines older persons as those aged over or equal to 60 or 65 years; now, over 703 million people are aged over 65 years, and that number is projected to double by 2050 [23]. Moreover, about 2 out of 3 older adults suffer from multiple chronic diseases [24], a condition to which mHealth proves to be a highly possible solution [3-7]. A myriad of frameworks and techniques have been employed to ensure the success of mHealth development and implementation with varying outcomes. Suggestions from research up to date stress the importance of having an in-charge multidisciplinary team working together with real end users rather than giving them the finished product out of the blue [25]. The International Organization for Standardization (ISO) 9241-210 further elaborates this concept in the term “human-centered design” (HCD) as the “approach to systems design and development that aims to make interactive systems more usable by focusing on the use of the system and applying human factors/ergonomics and usability knowledge and techniques”, in which the word “human-centered” is used to highlight that the process includes all stakeholders and not just the users [26]. Thus, in this review, the term “user centered” will be referred to as “human centered” to reflect its definition better.

### Review Objective and Question

In searching for the best methodology to create the most usable mHealth, many have put the said value at the core of their work: having the humans at the center of focus. This review aims to explain how HCD can be applied to create mHealth suitable for older adults and to summarize the overall process with recommendations from relevant primary research studies of mHealth design and development.

The research question of this review is the following: How can HCD be used to create mHealth solutions for older adults? This issue was formed during the first author’s attempt to develop an mHealth app for older adults to solve their current pain points in a geriatric wellness clinic. Despite the constant mentioning of HCD, previous scoping searches of literature bear a heterogeneous group of research studies differing in interpretation, execution, and the extent of evaluation. The need for further clarification on the procedural details is identified.

## Methods

### Design

A systematic approach following Siddaway et al’s guide [27] was employed to ensure a robust acquisition of the existing literature related to the topic with a method as reproducible, transparent, and unbiased as possible. The review was conducted following the Preferred Reporting Items for Systematic Reviews and Meta-Analyses (PRISMA) statement [28] (Multimedia Appendix 1). Detailed methods are described in the review as no prior protocol was published.

### Eligibility Criteria

Textbox 1 presents the eligibility criteria. As this review aims to draw from studies of a relatively new and emerging subfield of study, the criteria are inclusive. However, a certain degree of clarity in participants, qualitative or quantitative methods, analysis of the results, and discussions of the implementation results are required. Moreover, to best answer the review question, the included studies have to have these 3 key steps starting from (1) designing solutions based on existing problems, (2) developing the designed solutions, and (3) evaluating the developed solutions, all stated to be conducted in accordance with the HCD philosophy.

**Eligibility criteria.**

**Inclusion criteria**

- Community, primary, secondary, or tertiary care.
- Any qualitative, quantitative, or mixed methods study of original primary research.
- Participants must include, but not limited to, older adults (aged ≥60 years).
- Design goals must focus on mobile health (mHealth) solutions in the form of mobile apps intended for older adults.
- Study procedures must be in line with the human-centered design (HCD) philosophy.
- Studies must include details of mHealth apps and their development process, participants, design goals, and some implementation data.
- Studies depicting different processes of the same product/project are included. For example, an mHealth project might have 2 separate papers such as 1 for design and 1 for evaluation; both are included in this review.
- Trial and pilot studies are included.

**Exclusion criteria**

- Non-English language papers.
- Any type of literature review, narrative review, or systematic review.
- Studies with no relevant data or information that is of interest to the review question.

### Search Strategy and Study Selection

Systematic searches were conducted from the following 4 databases: IEEE Xplore, Medline via Ovid, PubMed, and Scopus. To best ensure comprehensive search results, search strings were compiled from keywords of the review question. Listed below are those strings with their corresponding similar terms:

- “mHealth” OR “mobile health”, for the app to be reviewed;
- “human centered” OR “human centered” OR “user centered” OR “user centered”, the approach in question;
- “design” OR “development”, the process required;
- “usability”, an outcome of HCD according to ISO 9241-210;
- “elderly” OR “older adults” OR “geriatric”, the target population.

Each group of strings was put together with the “AND” Boolean operator in the search engines as all of the above key terms were required by the set eligibility criteria. No date range was set. Manual searches on Google Scholar and the references of the eligible papers were also conducted to identify possible additional relevant papers for screening. All searches were performed by a single reviewer (ZN) on the same day (November 12, 2020). The reason why the ACM Digital Library was not included is discussed in the “Limitations” section.

Microsoft Excel was used to record and manage the search results; duplications were removed. Two independent reviewers (SB and ZN) screened the deduplicated results by titles and abstracts. The full-text screening was done by the same reviewers using the eligibility criteria from Textbox 1. The results were in agreement. The reviewers then proceeded to appraise the study qualities using the Mixed Methods Appraisal Tool (MMAT) for mixed methods studies [29]. Disagreements were resolved through discussions. As this review aimed to be inclusive, study quality was not used to exclude any paper from the review but rather to inform about the present research quality of the existing literature of interest. We chose MMAT as our appraisal tool because (1) it can appraise the heterogeneous methodologies of design studies and (2) its methodological focus helps reflect on the existing research critically. Table 1 presents the qualities of the included studies appraised by MMAT.

**Table 1:** Quality appraisal of included studies.

Studies	Criteria from the Mixed Methods Appraisal Tool														
	1.1^a^	1.2^b^	1.3^c^	1.4^d^	1.5^e^	4.1^f^	4.2^g^	4.3^h^	4.4^i^	4.5^j^	5.1^k^	5.2^l^	5.3^m^	5.4^n^	5.5^o^
Cornet et al [30]	1	1	1	1	1	N/A^p^	N/A	N/A	N/A	N/A	N/A	N/A	N/A	N/A	N/A
Cornet et al [31]	1	1	1	1	1	0	0	1	0	1	1	1	0	1	0
Fortuna et al [8]	1	0	0	1	1	0	0	0	0	1	0	0	0	0	0
Harte et al [32]	1	1	1	1	1	0	1	1	0	1	1	1	1	1	1
Harte et al [33]	1	1	1	1	1	N/A	N/A	N/A	N/A	N/A	N/A	N/A	N/A	N/A	N/A
Petersen et al [34]	1	1	1	1	1	0	0	1	0	0	1	1	1	1	0
Srinivas et al [35]	1	1	1	1	1	0	0	0	0	1	0	1	1	1	0
Stara et al [36]	1	1	1	1	1	N/A	N/A	N/A	N/A	N/A	N/A	N/A	N/A	N/A	N/A

^a^Is the qualitative approach appropriate to answer the research question?

^b^Are the qualitative data collection methods adequate to address the research question?

^c^Are the findings adequately derived from the data?

^d^Is the interpretation of results sufficiently substantiated by data?

^e^Is there coherence between qualitative data sources, collection, analysis, and interpretation?

^f^Is the sampling strategy relevant to address the research question?

^g^Is the sample representative of the target population?

^h^Are the measurements appropriate?

^i^Are the confounders accounted for in the design and analysis?

^j^Is the statistical analysis appropriate to answer the research question?

^k^Is there an adequate rationale for using a mixed methods design to address the research question?

^l^Are the different components of the study effectively integrated to answer the research question?

^m^Are the outputs of the integration of qualitative and quantitative components adequately interpreted?

^n^Are divergences and inconsistencies between quantitative and qualitative results adequately addressed?

^o^Do the different components of the study adhere to the quality criteria of each tradition of the methods involved?

^p^N/A: not applicable.

### Data Extraction

One independent reviewer (ZN) performed data extraction from the 8 eligible papers. The information from 5 mixed methods studies included (on the data extraction form) the year of the study, the country of the study, the name of the project (if stated), study design, design goals, participants, study methods, quantitative or qualitative data used, results, and key discussions. The information from the other 3 case studies included the year of the study, the country of the study, goals, and results. All extracted texts were manually typed in Microsoft Excel.

### Synthesis of Results

Because of the heterogeneous nature of the included studies, narrative synthesis was chosen. Following Popay et al’s guide [37], the narrative data synthesis was performed iteratively between the 4 key elements as explained in Textbox 2.

**Key elements for the narrative data synthesis.**

- Developing a theory of how the intervention works, why, and for whom

Previous studies were carried out under the same hypothesis that human-centered design (HCD) helps make a more usable system for its users. This review adopted that same assumption and aimed to elaborate on how HCD works, especially for older adults, in steps.

- Developing a preliminary synthesis of findings of included studies

Textual descriptions together with tabulation were chosen to summarize and display the extracted data. A recurring concept was identified across the studies: the HCD process. To ensure transparency, suggested HCD activities from ISO 9241-210 were chosen to categorize these patterns into 4 steps as follows: (1) understand and specify the context of use, (2) specify the user requirements, (3) produce design solutions to meet these requirements, and (4) evaluate the designs against requirements [26].

- Exploring relationships in the data

Qualitative case descriptions were used to explore details and findings among included studies that correlate with each theme/step. A conceptual diagram was then created to answer the review question. The diagram was structured according to the Minto pyramid principle, using the following rules: (1) ideas at any level in the pyramid must always be summaries of the ideas grouped below them, (2) ideas in each grouping must always be the same kind of idea, and (3) ideas in each grouping must always be logically ordered [38].

- Assessing the robustness of the synthesis

All included studies were appraised by Mixed Methods Appraisal Tool (MMAT), and the synthesis process was reflected on critically.

## Results

### Study Selection

**Figure 1:**
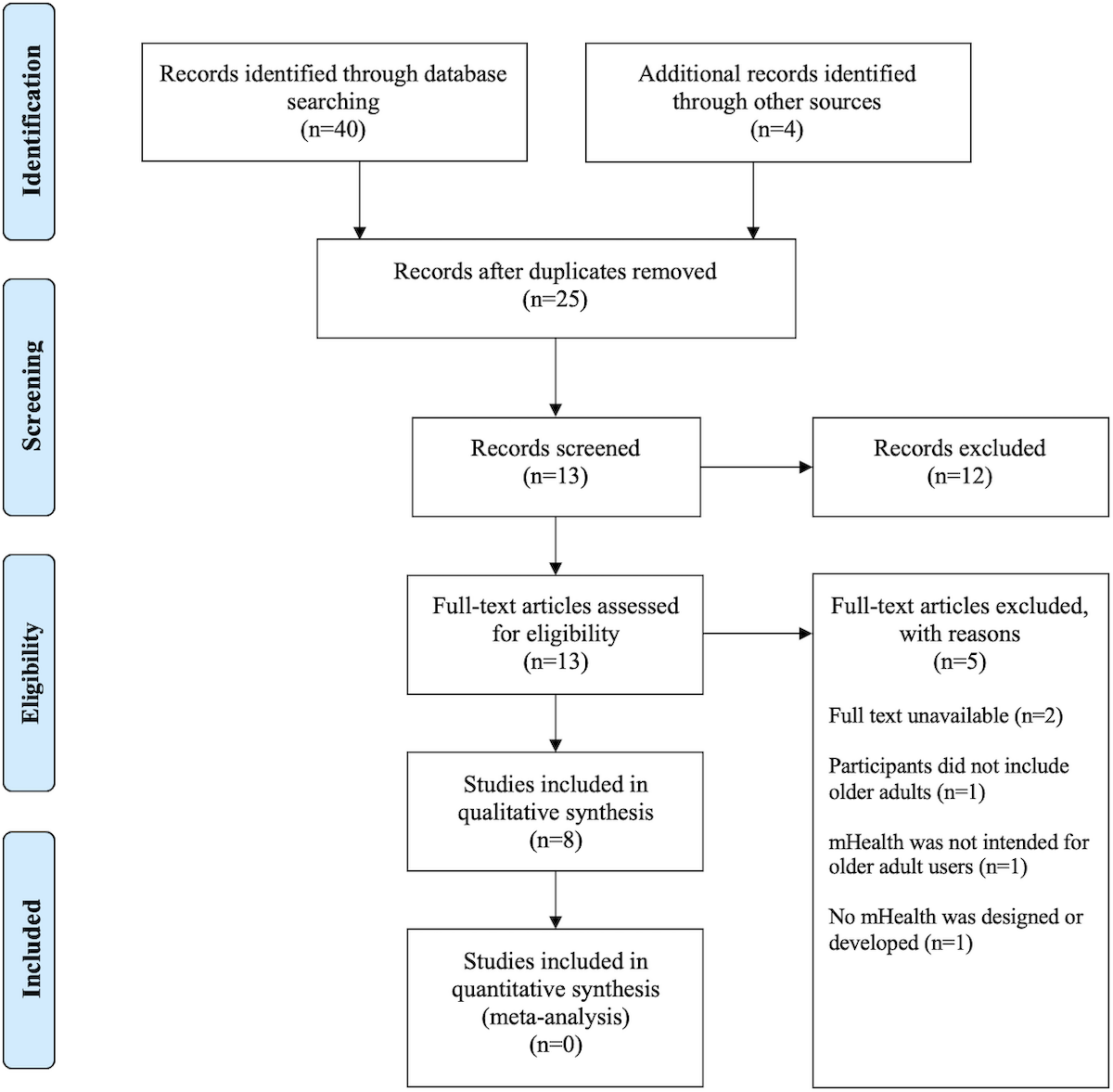
PRISMA flow diagram. mHealth: mobile health; PRISMA: Preferred Reporting Items for Systematic Reviews and Meta-Analyses.

Figure 1 shows the selection process of the included studies. The initial search yielded 44 studies, of which 40 were from the 4 databases and the other 4 were from Google Scholar. A total of 25 studies remained after the removal of duplications. Two independent reviewers (SB and ZN) screened titles and abstracts according to the criteria. The remaining 13 full-text studies were then assessed by the same 2 separate reviewers for eligibility. Five studies were excluded, as shown in Figure 1. Eventually, 8 studies were retained for this systematic review.

### Study Characteristics

Five studies were mixed methods, employing both quantitative and qualitative techniques to design and evaluate mHealth apps. Three were qualitative case studies focusing on describing the methodology and problems found during the process with little or no actual quantitative or qualitative data from the research shown. One of the 3 case studies was about the same process from the same project of another included mixed methods study; it was included for its qualitative reflections on the design and development processes. All studies stated clear aims of the research and were deemed relevant to this review question.

To illustrate the overall quality of the included studies, their quality appraisal scores were reported in the MMAT-suggested format [39] in Table 1. Of the 8 included studies, all were rated to have adequate quality in their qualitative part. However, only 1 study had a passable rating of 60% in its quantitative part, while the rest were rated poor. The quantitative criteria that all studies failed were about the sampling strategy and the account for possible confounders. The description of the processes was lacking, leading to questionable results and interpretation. This issue was further explored in the narrative synthesis of results.

Table 2 summarizes HCD processes of the 5 included mixed methods studies. Four of the studies were conducted in the United States, with only 1 study conducted in Ireland. Two studies under the same project focused on patients with heart failure [31,35]. One study aimed at patients with psychiatric disorders [8]. The other 2 studies dealt with fall risk assessment and detection [32] and sarcopenia prevention [34], respectively. In addition, of the 3 included qualitative case studies, 2 reflected on the same project as the mixed methods study dealing with falls in Ireland [33,36]. By contrast, the remaining 1 study reflected on a different project targeted at patients with cardiac implantable electronic devices (CIEDs) in the United States [30].

**Table 2:** Summary of the included mixed methods studies.

Study	Project	Setting	Design goal	Participants	Methods
Cornet et al [31]	Engage	Academic health center, the United States.	To evaluate and test the usability of a self-managing heart failure system for older adults developed in a study by Srinivas et al [35].	(1) 13 older adults and (2) 2 caregivers	Study I: (1) A structured interview was used to assess participants’ daily self-management routines and technology familiarity. (2) The think-aloud method was employed as each participant completes 8 given tasks on the system. (3) Feedback from the patients after they finish was used. Study II: (1) The system was re-designed after Study I. (2) A structured interview was used. (3) The think-aloud method was employed as each participant completed a given scenario in which he/she was to act as if he/she were the assigned fictitious character. (4) Feedback from the patients after they finish was used. (5) SUS^a^ was used after usability evaluations. (6) NASA-TLX^b^ was used after usability evaluations.
Fortuna et al [8]	—^c^	Specialized center, the United States.	To incorporate an existing psychosocial intervention into a selected mobile platform.	Phase I and Phase II: (1) authors; Phase III: (1) older adults and (2) experts; and Phase IV: (1) 10 middle-aged and older adults	Phase I: (1) A literature review was done to identify requirements. Phase II: (1) A literature review was done to find a suitable existing mobile platform. Phase III: (1) The interdisciplinary panel of end users and experts work together to incorporate an existing psychosocial intervention into the chosen mobile platform. Phase IV: (1) The think-aloud method was employed as each older adult goes through task-based usability testing. (2) Feedback from patients was collected. (3) Surveys based on SUS; Post-Study System Usability Questionnaires; and USE^d^ questionnaires were used after each usability testing. (4) The ability to perform tasks without help was recorded in percentage.
Harte et al [32]	Wireless Insole for Independent and Safe Elderly Living	Academic health center, Ireland.	To develop, assess, and enhance usability and user experience of a mobile app of a connected health system designed for fall risk assessment and fall detection.	Phase I: (1) 10 experts and (2) 12 older adults; Phase II: (1) 10 experts from Phase I; and Phase III: (1) 10 older adults from Phase I	Phase I: (1) Likert scales were used to rate mock-ups called use cases. (2) The think-aloud method was employed during use case analysis. (3) Self-reported measures of the experts were collected. (4) Visual perception and cognitive processing metrics of older adults were collected. Phase II: (1) Likert scales were used to rate the paper prototypes based on use cases by experts. (2) ASQ^e^ and chosen usability metrics were used to rate the developed mobile working prototypes by experts after scenario-based usability testing. (3) The think-aloud method was employed during experts’ mobile working prototype runs. Phase III: (1) Likert scales were used to rate the mobile working prototypes by older adults. (2) ASQ, SUS, NASA-TLX, and chosen usability metrics were used to rate the mobile working prototypes by older adults after scenario-based usability testing. (3) The think-aloud method was employed during older adults’ working prototype runs.
Petersen et al [34]	—	Academic health center, the United States.	To create a mobile app for older adults to monitor their use of a Bluetooth-connected resistance band for sarcopenia prevention.	Round 1: (1) 6 older adults; Round 2: (1) 3 clinicians and (2) 4 older adults; Round 3: (1) 3 clinicians and (2) 6 older adults	Round 1: (1) Semistructured interviews gave information on how the app can be of use. Round 2: (1) The think-aloud method was employed as participants go through the wireframes. (2) A verbal prompting method was employed to encourage participants to give their thoughts. (3) Oral feedback from participants was recorded as they go through the video contents to be used in the prototype app. (4) The SUS was used after each participant finishes. (5) The USE score was used after each participant finishes. Round 3: (1) The think-aloud method was employed as participants go through the wireframes. (2) A verbal prompting method was employed to encourage participants to give their thoughts. (3) The SUS was used after each participant finishes. (4) The USE score was used after each participant finishes.

Srinivas et al [35]	Engage	Specialized center, the United States.	To design, develop, and evaluate a consumer-facing health information technology system that supports heart failure self-care.	Phase I: (1) 63 older adults, (2) 35 caregivers, and (3) additionally data on 66 patients obtained from other literature; Phase II: (1) experts; Phase III: (1) 1 expert and (2) 5 older adults	Phase I: Major themes of the app were synthesized from data gathered through direct observations at patient outpatient visits, standardized surveys on patient self-care, patients’ electronic medical record reviews, and semistructured interviews focused on patient self-care. Phase II: Core activities of the app were determined through educating, brainstorming, and design sessions of the research team. Phase III continues in Cornet et al (2017) [31]: (1) heuristic evaluation done by the team’s expert identified and classified usability flaws. (2) Structured interviews focusing on patients’ self-care routines were done before usability testing. (3) The think-aloud method was employed during laboratory-based usability testing of the developed prototype as each older adult goes through the tasks given on a mobile. (4) Questionnaires adapted from the SUS were used after each usability testing.

^a^SUS: System Usability Scale.

^b^NASA-TLX: NASA-Task Load Index.

^c^Not stated.

^d^USE: Usefulness, Satisfaction, and Ease of Use.

^e^ASQ: After Scenario Questionnaire.

### HCD Activities in mHealth Development for Older Adults

#### Overview

All 5 mHealth projects, from the included 5 mixed methods studies and 3 case studies, have the 4 key steps from ISO 9241-210 in their HCD processes, albeit described and mentioned to varying degrees. This section explores and illustrates these recurring steps across all included studies using the qualitative case description technique. All 8 studies are summarized and described in 4 HCD steps. Each step has 5 paragraphs representing a total of 5 mHealth projects: the first for patients with heart failure [31,35], the second for patients with psychiatric disorders [8], the third about falls in the elderly [32,33,36], the fourth for sarcopenia prevention [34], and the fifth about CIEDs [30].

#### Step 1: Understand and Specify the Context of Use

Understanding the context of use such as the end users, their current tasks, key activities, and working environment is essential to the design process; it helps guide how solutions should be tailored and set practical goals for the project [26].

Srinivas et al [35] used various HCD frameworks to develop an mHealth app that helped older patients with heart failure to improve their self-care engagement, health behaviors, and knowledge of the disease. In 2 years, the researchers collected data from 65 older patients with heart failure and 35 caregivers through direct observations at outpatient clinics, electronic medical record reviews, and semistructured interviews; the patients’ health care routines, health literacy, environments, and supports were the priority. They conducted these field-based investigations in an academic medical center in Southeast United States. In addition, the authors included 66 other patient data from the United States and Singapore in an urban emergency setting. Details on the sampling method and rationales for the number of patients were not provided. It was also noted that not all data could be utilized fully in the design process. The qualitative quality of this study was adequate.

Fortuna et al [8] aimed to integrate self-management intervention into a mobile app for middle-aged and older patients with psychiatric disorders to promote self-care for better health outcomes. However, rather than obtaining data directly from potential users, the researchers gathered rationales and pain points of the project from a literature review. Details about the method were not specified in the paper. No quality appraisal of the included literature was presented. They then used the review results in the subsequent design. For example, integrating an existing intervention to an existing mobile platform was chosen over developing a new one because it was more practical. Characteristics specific to the elderly such as declining cognitive functions affecting their self-management and motivation, multimorbidity, and limited digital literacy were considered. The researchers also decided the intervention to be implemented based on the literature review: Integrated Illness Management and Recovery (I-IMR), an evidence-based medical practice for psychiatric patients, was cited to be promising and thus chosen. The authors reported successful implementation and noted that identifying the unique needs of the intended users to guide the design process helped build a more usable product. The qualitative methodologies were appraised to be of adequate quality.

Stara et al [36] integrated HCD into the development process of their connected health system: the Wireless Insole for Independent and Safe Elderly Living (WIISEL), consisting of a pair of chargeable insoles with Bluetooth transmission, its charger, a smartphone app, a gait analysis desktop application, and an administrative web application. The authors drafted a preliminary concept of the system and then discussed it in 3 focus groups with 6 older adults and 6 stakeholders in each group; the focus groups were conducted at 3 separate sites: a primary care center in Ireland, a tertiary care center in Israel, and a specialized center in Italy. The sampling method was not specified. The qualitative quality is adequate. The authors concluded that barriers to technology-enabled care acceptance in older adults were related to security, intrusiveness into their home environment, lack of control, confidentiality, and usability issues worsened by aging. Thus, involving users early in the process proves vital in crafting a health care technology that matches actual older adult user needs, with elderly friendly user interfaces and safety being a priority.

Petersen et al [34] used HCD to develop an mHealth app featuring exercise videos to work with a Bluetooth-connected resistance band that together would help health care providers monitor older adults’ exercise progress for sarcopenia prevention. A convenience sampling method was used. Six older adults were recruited from a primary care clinic at an academic health center in the United States. The researchers then conducted semistructured interviews to assess the patients’ general views regarding mHealth, their current activities, and their opinions of the Bluetooth-connected band and sample exercise videos. They further explored the participants’ opinions in using technology to help with their exercise therapy. The quality of the study was appraised to be adequate. The participants had positive responses to the idea. All had experience using smartphones. Notes from these interviews were then used as key information to guide further design processes.

Cornet et al [30] implemented HCD in developing an mHealth app that shared the information stored in CIEDs of patients with heart failure with the patient themselves. In 3 months, 24 older patients with heart failure, 12 of whom had CIEDs, were recruited from a major health system in the Midwestern United States for semistructured interviews to gain context about their health decision-making processes. No sampling method was stated. The interview utilized 2 notable approaches: (1) the critical incident technique, which involves asking the interviewee to recall a particular past event to gain insights through their actions and experience at the time; and (2) the think-aloud method, which lets the interviewee talk about what he/she was currently doing or would do in a given event. The researchers then analyzed and synthesized the gathered data into 2 outputs: (1) personas, a design technique that groups users based on their behaviors; and (2) use-case scenarios (or as-is scenarios), another design technique that depicts how users make decisions in hypothetical situations. These outputs were then cross-checked with 2 patient advisors, older adult patients who volunteered to help with design, and a group of 7 clinician experts from the same major health system. The patient advisor meeting was held early to gain additional inputs and feedback to help the team make more relatable personas and use-case scenarios. The clinician meeting was held later and focused more on the validity and feasibility of the subsequent processes. Besides, direct observations at the CIED clinic and meeting with 2 cardiologists were also done with the same objectives. The methods were appraised to be adequate in quality. As the paper is a case study, challenges and recommendations by the authors were reported. First, logistics issues including but not limited to compensation, conflicts of interests, older adult limitations, patient data, recruitment criteria, and stakeholder meetings need to be addressed or consulted with professionals to ensure efficiency and efficacy. Second, stakeholders should be involved early in the design process, and their roles should be identified clearly in how active they would be; for example, it might just be getting informed about the process, giving their opinions to the team, or having specific tasks given to them. The authors also added that more roles are not always better, and stakeholder involvement should be carefully balanced. Third, an adequate recruitment method should be employed to secure a representative group of potential users. Also, a selection of stakeholders who work well with the development team is key. Fourth, direct and timely communication between development team members and relevant stakeholders is recommended, although it might be difficult to achieve at times.

#### Step 2: Specify the User Requirements

The second step of HCD focuses more on synthesizing further outputs from the first step. The goal is to derive what the users need to do and their objectives based on the gathered context and then set a clear statement of user requirements for the solution designs [26]. User requirements lay down the groundwork for how the product should be created and which performance or criteria should be measured to evaluate the product. These requirements are often created along with other requirements of the product such as the requirements of the system stating that the system needs to be able to do a certain task because it will help users accomplish their goals.

Srinivas et al [35] reported a successful translation of major themes from the gathered data and created a set of requirements for the subsequent design. Thematic analysis was done to identify user needs; it was concluded that the patients lack adequate health information and communication regarding their conditions and disease progresses, they are disengaged from their self-care due to the added burden, and they are not equipped with practical knowledge nor tools for optimal self-management. The authors then held educating sessions with the design team, composed mainly of experts from technical and HCD backgrounds, on the phenomena of interest through various media and means from the collected data. Next, brainstorming sessions were held. The requirements were derived from the previously identified major themes from the research: the system needed to be viewable by the patients and potentially their health care providers, simple to use, complementing well with their self-care routines, and customizable. Details of the performance goals to be evaluated were not stated.

Fortuna et al [8] derived requirements from their literature review of user interfaces for older adults, I-IMR contents, and the interdisciplinary panel of end users and experts recruited from the site where the project was intended to be launched. I-IMR is a clinical psychological intervention that requires both health care providers and patients to work together in 10 training modules/sessions covering 4 topics on psychoeducation, behavioral tailoring, relapse prevention training, and coping skills training over 8-10 months. Adapting this face-to-face intervention and its contents into a suitable mobile experience for middle-aged and older patients was key to the project. Details about the performance goals were not stated.

Stara et al [36] stated in their study that user requirements for the WIISEL-connected health system were defined by the design team together with 18 older adults who were potential users and 18 stakeholders who were geriatricians, neurologists, nurses, and physical therapists in 2 sessions of focus groups. Details of the participants and the final user requirements and performance goals were not provided.

Petersen et al [34] concluded pain points from their previous research regarding the exercise videos as follows: specific movements were hard to identify from low-contrast backgrounds, and instruction sounds were not heard clearly. Participants also stated that big and clear repetition numbers would help them better keep track, feedback and instructions would help them finish the exercises at home, and tablets were preferred as they have large screens. The authors did not show an explicit statement of user requirements or performance goals in the paper.

Cornet et al [30] did not provide details regarding the process of writing user requirements in their case study.

#### Step 3: Produce Design Solutions to Meet These Requirements

This HCD step focuses on designing how the users interact with the system based on the requirements from the previous step [26]. HCD strived for the best user experience. The process needs to be iterative and flexible to address user needs and requirements that are often hard to identify completely in 1 cycle. The outputs from this step are also used to explain and communicate the design concepts with stakeholders, simulate possible scenarios of its uses, and ultimately specify how the system is to be developed.

Srinivas et al [35] created design solutions from the requirements specified. The team members raised diverging ideas from those requirements. They then worked together to converge those ideas into 4 main potential design solutions: (1) a short-term intervention of 30 days to encourage user adoption of the system, (2) an avatar representing the results of different self-care routines to teach users about cause and effect in a more engaging way, (3) a function that allowed users to set and keep track of their goals to promote health behaviors, and (4) a tool that helped enhance clinical visit experience to improve communication and collaboration with their health care providers. Finally, the team decided to develop the mHealth solution based on the 30-day intervention idea. The system was to have 3 main modules to serve all user requirements previously set: (1) LOG, for users to log their health information; (2) HINT, a collection of short materials about heart failure disease and self-care; and (3) GOAL, gamified daily goals for better self-care behaviors. User–system interaction and user interfaces were then developed in subsequent design sessions composed of 3-6 members of the design team. Paper prototypes, Microsoft PowerPoint wireframes, and software prototypes were developed successfully. The authors noted that although clinical experts were consulted from time to time during this step, stakeholders, including end users, did not really participate in the design process; logistics issues and not knowing how to involve older adults were accountable for this approach. The authors also added that their waterfall approach to development, meaning the design process was linear and required time before evaluation, caused delays in solving design problems that could be prevented if a more agile approach was adopted.

Fortuna et al [8] created scenarios of uses, user–system interaction, and user interfaces based on the identified user needs and requirements. The system was designed to have the following: an ability to be customized, a tracking system to show users their progress, a monitoring system to send data back to health care providers, and a messaging system from health care providers for more human interaction and a smoother workflow. The text contents from I-IMR manual, originally intended for clinicians, were also modified to fit smartphone pages and rewritten using the Flesch-Kincaid Grade Level formula in Microsoft Word to a simpler sixth-grade level. The 4 core topics of I-IMR were also re-designed to fit the mobile experience. Psychoeducation used short videos that showed clinicians teaching self-management techniques to patients to help users master the skills. Behavioral tailoring utilized educating modules, a medication schedule function, and a reminder system to make patients take their medication on time. Relapse prevention training, usually done by exploring a patient’s experience and identifying triggers to create a prevention plan for a possible relapse, offered an already made plan that was accessible and editable on mobile at any time. Coping skills training also used videos as media to equip users with the tools to help them in the real world, for example, relaxation videos that guide users to self-soothe and calm themselves down. In addition, issues regarding data security and mHealth user disengagement were addressed: the project adhered to Health Insurance Portability and Accountability Act (HIPAA) compliance and involved health care providers to encourage adoption.

Stara et al [36] stated in their study that the subsequent design process of the smartphone app of the WIISEL system is elaborated in 2 studies included in this review: 1 mixed methods study focused on the findings by Harte et al [32] and the other case study focused on the HCD methodology also used by Harte et al [33,36]. Rapid development was employed to create, test, and produce 4 versions of prototypes, 2 on paper and 2 on mobile. The first paper prototypes (also called “use cases”) consisting of scenarios of use, descriptive end user profiles, storyboards, and interface mock-ups were created from the opinions of all project stakeholders. These use cases were based on key activities that the users needed to carry out. Ten multidisciplinary experts and 12 older adults were then recruited by a purposive sampling method to analyze these use cases. This analysis quickly identified usability problems that were in turn fixed by the development team. The first mobile prototypes together with user manuals and the updated, second paper prototypes were then produced. The manuals were created to help address usability problems that could not be fixed by the development team such as the built-in buttons, the operating system keyboard design, the impracticality of an automatic data sync, and the connection limitations. The same experts then evaluated the first mobile prototypes simultaneously with the guide of the second paper prototypes and the user manuals. The results from this second expert analysis were then used to design the second mobile prototype for another usability test with end users. The authors added that multiple inputs from the relevant stakeholders, although divergent in nature, are essential to HCD and could be obtained only with enough rounds of iterations. Thus, the rapid cycles of using paper prototypes and expert evaluation for fast feedback before end user usability testing are recommended.

Petersen et al [34] updated the exercise videos from user pain points and created mobile prototypes as black and white wireframes showing simple outlines of the designed user interfaces. The researchers then presented exercise videos and the wireframes with different design approaches to 3 clinicians and 4 older patients from an academic health center in the United States for additional inputs. The pain points gathered from the older patients about the videos and the wireframes were as follows: the video instructions should be slower and have more details, the videos should have subtitles with large fonts, the video sound frequency should be adjustable, and the progress bars in the wireframes should be vertical. By contrast, the clinicians were content and also suggested the use of the Borg Scale of Perceived Exertion to measure each exercise difficulty in comparison to the others. Finally, the team created the interactive mobile prototypes featuring playable updated exercise videos with clear instructions and the colored user interfaces for usability testing in the next step. The authors noted that health management is a process that needs both health care providers and patients; therefore, the more stakeholder groups involved, the more complete the design of the mHealth that aims to assist the process.

Cornet et al [30] produced and improved 4 versions of prototypes through iterative prototyping and testing. The mHealth app for patients with heart failure with CIEDs was designed to have 4 main features: a heart health score derived from CIED data for the patient audience, self-assessments covering topics of recommended self-care routines, guides for better heart failure self-management, and logs showing data from CIEDs. The first prototype design was reported to take 5 months to complete. The 2 patient advisors from the research phase helped review this early prototype design. The later 3 prototypes then took 2, 1, and 3 months, respectively, with the final prototype going through refinement for heuristic evaluation for another 1 week. Feedback from each prototyping and testing was used to improve the later versions. More details of the process were not stated in this case study; however, challenges and recommendations were reported. First, the authors found that design solutions should be based on evidence gathered from the potential users of the project to avoid bias or assumptions of the design team. This challenge benefits from stakeholder involvement and rapid cycles of testing and feedback. Second, design solutions and features should be prioritized and focused on. Grouping these solutions into modules and structurally planning how to develop and test them to determine what works and what does not could help simplify the process. The third point elaborates on the first point but focuses on the feasibility of the proposed design solutions: to balance the design team’s creativity with practicality. All limitations or regulations regarding the mHealth and its implementation should be worked out properly with the stakeholders to avoid project failure.

#### Step 4: Evaluate the Designs Against Requirements

The human-centered evaluation activity is vital to HCD and is iterative by its nature [26]. As illustrated in the third step, producing design solutions usually follows by evaluating them to assess their abilities to fulfill the requirements, obtain user feedback, gain more user needs, and quantify the results as baselines or for comparisons.

Srinivas et al [35] conducted a series of evaluations on the wireframes and the prototypes in parallel with the design process. First, heuristic evaluation by the team’s human–computer interaction expert guided by Nielsen’s usability heuristics [40,41] was done before the software prototype development. This helped transition the static wireframes into the interactive software prototypes and identify usability flaws early in the process for correction. The authors reported 45 flaws, of which 6 were major flaws. The corrected software prototypes were then evaluated by older adults and caregivers as elaborated further in another study by Cornet et al [31]. The researchers conducted the evaluation in 2 phases in a laboratory setting: (1) a task-based usability test with 5 users and (2) a scenario-based usability test with 10 users. A total of 13 patients with heart failure and 2 informal caregivers aged over or equal to 60 years were recruited from an urban and another suburban outpatient cardiology clinic of an academic health system in the Midwestern United States. All consented to the study and were compensated with US $40 gift cards. Details of the sampling method were not specified. All participants were given mobile devices with the software prototypes installed ready for testing. Both tests involved structured interviews about users’ self-care routines and familiarity with technology at the start, the think-aloud method by talking out loud about what they were thinking during testing, and the use of standardized evaluation tools at the end. The tools include (1) System Usability Scale (SUS) consisting of 10 questions about the overall usability of the product and reporting in a score of 0-100 with 68 defined as average usability [42] and (2) NASA-Task Load Index (NASA-TLX) consisting of 6 scales to assess the cognitive load expended during product use [43]. SUS was used in both phases, but NASA-TLX was used only in the second phase. The tests were video recorded. The software prototypes were updated between the 2 usability tests. It was reported that SUS rating improved in the second phase from below average to above average. However, the authors stated that the result could be affected by the design changes made, the different usability testing methods, and the sampling techniques. Moreover, the wording of SUS was shown to be difficult to understand to a certain group of older adults and might not reflect the real usability of the system [44]. The methodologies were appraised to be of inadequate quality. The authors added that quantitative results from the standardized tests did not capture the whole picture of the system usability issues and should be interpreted together with the qualitative results. Some older adults also showed resistance toward these usability techniques, that is, the think-aloud method was strange and the fictitious event of the scenario-based testing was counterintuitive as they had to remember the mock details that were irrelevant to them and got distracted. Logistics issues such as the locations of the testing sites, the set ups of recording tools, and the transportation of the older participants also need to be addressed.

Fortuna et al [8] conducted 2 cycles of task-based usability testing with 2 different groups of 5 participants each. The authors deemed a minimum of 5 participants could identify most usability issues [45]. All participants were middle-aged and older patients with both medical and psychiatric illnesses recruited from 2 mental outpatient programs in New Hampshire. A purposive sampling method of reviewing medical charts and reaching out to potential patients for informed consent was used. Gift cards worth US $20 were provided upon participation. The participants were given mobile devices with the app installed and a list of tasks to complete. They were orientated on how to use the devices and what the think-aloud method was. The researchers also asked the participants about the user interfaces and assigned them adapted surveys based on SUS, Post-Study System Usability Questionnaire (PSSUQ), and the Usefulness, Satisfaction, and Ease of use (USE) questionnaire. PSSUQ is an 18-item questionnaire with 7 rating scales and 1 not-applicable rating, assessing user satisfaction with the system [46]. The USE questionnaire also contains multiple items with 7 rating scales that explore 3 dimensions: usefulness, satisfaction, and ease of use [47]. All sessions took approximately an hour and were audio recorded if allowed or noted in detail if not. Updates on the app were made between the 2 cycles from user feedback: the text and video contents were shortened and the reading level was reduced from sixth grade to fourth grade. The authors reported that all participants could finish given tasks independently and both the qualitative comments and quantitative surveys had positive results, suggesting the users were satisfied with the app and would continue to use it if encouraged to do so. The mixed methods methodology was appraised to be inadequate in quality. The authors remarked that (1) future behaviors, that is, whether the patients would use the app in a real-world environment or not, were hard to predict from 1 hour of usability testing in a controlled environment, (2) the patients recruited specifically for the purpose of usability testing might lack heterogeneity and did not fully represent the intended vulnerable group of interest, and (3) technology constraints of utilizing an existing platform were reported. The authors also added the results might prove relevant and beneficial to the research of a similar fashion, and more studies on the mHealth intervention effectiveness were needed.

Stara et al [36] incorporated evaluation early in their HCD process as shown in 2 studies by Harte et al [32,33]; the authors then conducted user testing of the finished system with 54 older users [36]. During the course of producing design solutions, evaluation was done on (1) the first paper prototypes or the use cases, (2) the second paper prototypes, (3) the first mobile prototypes, and (4) the second mobile prototypes. The participants were 10 multidisciplinary experts and 12 older adults recruited using a purposive sampling method. Self-reported measures regarding the experts’ knowledge together with the older adults’ visual perception and cognitive processing metrics were reported. For the first paper prototypes, 10 experts and 12 older adults analyzed the prototypes by going through each use case. The think-aloud method was used to gather qualitative inputs, and Likert scales that asked the users to rate the user interfaces and task flows were used after each use case to obtain quantitative results. Likert scales are 5-point scale questionnaires that can be used to quantify user satisfaction; the question can be, for example, “I have no problems using the system.” Usability problems of the first paper prototypes were then identified from think-aloud transcripts and grouped according to a derived set of heuristics [48]; the problems were then given severity rating based on the results of the related use case Likert scores. The prototypes were then updated accordingly. For the second paper prototypes, the experts analyzed the updated use cases in the same manner again to compare them with the first ones: most usability problems were reported to improve. For the first mobile prototypes, scenario-based usability testing was done by the experts as if they had been first-time users. The experts were able to use user manuals during testing. The sessions were also video recorded. Think-aloud scripts together with After Scenario Questionnaire (ASQ), SUS, and 3 usability metrics (ie, time taken to complete task, errors made, and completion rate) were used to update the user manuals and the user interfaces. The ASQ is a 3-item questionnaire regarding ease of completion, time taken to complete, and support information of the system with a 7-point scale, where a lower score indicates greater satisfaction [49]. For the second mobile prototypes, both task- and scenario-based usability testing were done by 10 older adults. They had access to the user manuals during testing. The sessions were video recorded. Data were obtained using the same methods as the first mobile prototypes with the addition of posttest interviews about general impressions of the system and NASA-TLX. The authors reported that the system achieved acceptability among end users. This is the only included mHealth project that has adequate quality appraised by MMAT. The authors concluded in their study that (1) older adults needed clear feedback from the app due to technology unfamiliarity, but imposing feedback such as alerts or cautions should be used only when necessary to avoid anxiety for the same reason; (2) older adults were found to be uncomfortable with touchscreen keyboards, thus minimizing or simplifying them would be ideal; (3) standardized tests such as SUS, ASQ, and NASA-TLX proved to give concordant and valuable information regarding the system usability, but they should be interpreted together with the more objective metrics, such as time taken to complete task, errors made, and completion rate for more tangible results; (4) expert evaluation before end user usability testing was efficient and thus recommended; and (5) multiple inputs from different stakeholders, despite being divergent in nature, were essential to HCD. The process took 12 months to complete, of which the first prototypes took around 6 months; it was noted that interviewing and testing all the participants in the first phase were the causes of the long duration. Finally, 54 older users then tested the WIISEL system, both the mobile app and the soles, in Ireland, Israel, and Italy [36]. The usability testing had 2 stages: (1) the 3-day pilot stage had 15 participants test the system in a laboratory setting for a day and then at home for 2 days without specific instructions, and (2) the validation stage had 39 participants use the system at home for 14 days also without specific instructions. The participants completed the 12-item Quebec User Evaluation of Satisfaction with Assistive Technology questionnaire (QUEST) and SUS after each stage. Both had positive results. The authors did not detail the process as the paper was a case study. They concluded that technology acceptance was most affected by the system effectiveness but could also be positively influenced by proper user training and support.

Petersen et al [34] evaluated the wireframes and the prototype app using both qualitative and quantitative methods. Three clinicians from an academic health center in the United States participated; 6 older patients were recruited from a primary clinic of the same health center. A convenience sampling method was used. Think-aloud and verbal prompting methods were employed during testing to gather qualitative feedback from the participants. SUS and USE questionnaires were used after each participant finished testing the wireframes and the prototype app. The USE questionnaires comprise 30 questions asking about usefulness, ease of use, ease of learning, and satisfaction of the system with a 7-point scale to rate them. All sessions were audio recorded. Usability scores of the wireframes and the prototype app were calculated together and showed no statistically significant differences between the clinician and the patient participant groups with mean SUS scores of 65.8 and 66.8, respectively. The mixed methods study was appraised to be of inadequate quality. In addition, sentiment analysis of the participants’ recorded statements was done; its results were in accordance with the SUS scores. A further application of natural language processing–based Dirichlet allocation topic modeling of the recorded statements showed that clinicians and older patients had different topics of interest regarding the mHealth system. The authors concluded that (1) inclusion of different stakeholder groups was vital to HCD because each has a different perspective on the mHealth system as illustrated in the study, (2) sentiment analysis could prove useful to HCD by effectively and efficiently analyzing qualitative inputs alongside traditional usability techniques, and (3) future research on incorporating sentiment analysis and natural language processing in HCD was encouraged.

Cornet et al [30] conducted 3 usability evaluations and 1 heuristic evaluation concurrently with the iterative production of 4 prototypes. The 3 rounds of usability evaluations had 4, 8, and 12 older patients with heart failure as participants, respectively. Details of the sampling method were not specified. The software prototypes were installed on prepared smartphones for the tests. The first 2 rounds were 90-minute task-based usability testing. Participants had to complete demographic surveys and the Newest Vital Sign (NVS) health literacy screening before the test. NVS is a screening tool that takes 3 minutes to complete; it has 6 questions asking about a nutrition label that is given to the patient to assess their health literacy [50]. The think-aloud method was used during the test. Participants had to complete SUS, NASA-TLX, and user acceptance survey once finished. They were also interviewed about the system after the test. The third round was a 90-minute scenario-based usability testing simulating the use of the system in the first 10 days. The participants in this round were also required to have CIEDs. Data were gathered in the same manner as in the first 2 rounds. Finally, heuristic evaluation was done by 3 outside HCD experts for refinement of the system; the process took 2 weeks. Details of the results were not stated. However, the authors reported challenges and recommendations found as the results of their case study. First, laboratory usability testing is good for detecting general software issues (eg, user interfaces and navigation), but it might not be able to address real-world usability issues. Thus, system evaluation at the actual site of the intended setting should be considered as time and the budget allow. Second, standardized tests should be adapted to fit real users, for example, the word “cumbersome” in SUS was changed to “awkward” in the study as the older adults could understand it better. Third, the testing process tends to get complicated and lengthy with numerous tools and techniques employed, therefore the HCD team should opt to cut reducible workload, manage time between testing and analyzing, and look for the possibility of utilizing automated data collection or analysis.

## Discussion

### Principal Findings

**Figure 2:**
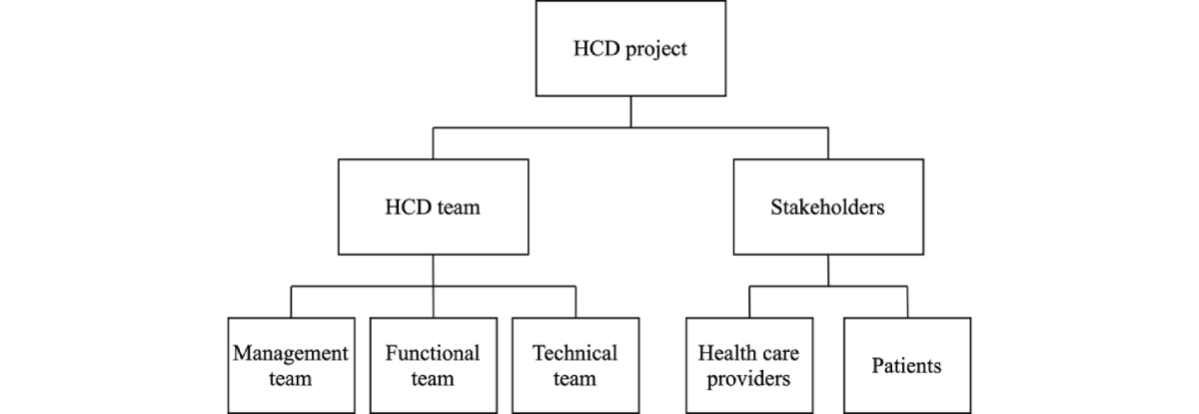
Pyramid model of HCD project by structural order. HCD: human-centered design.

**Figure 3:**
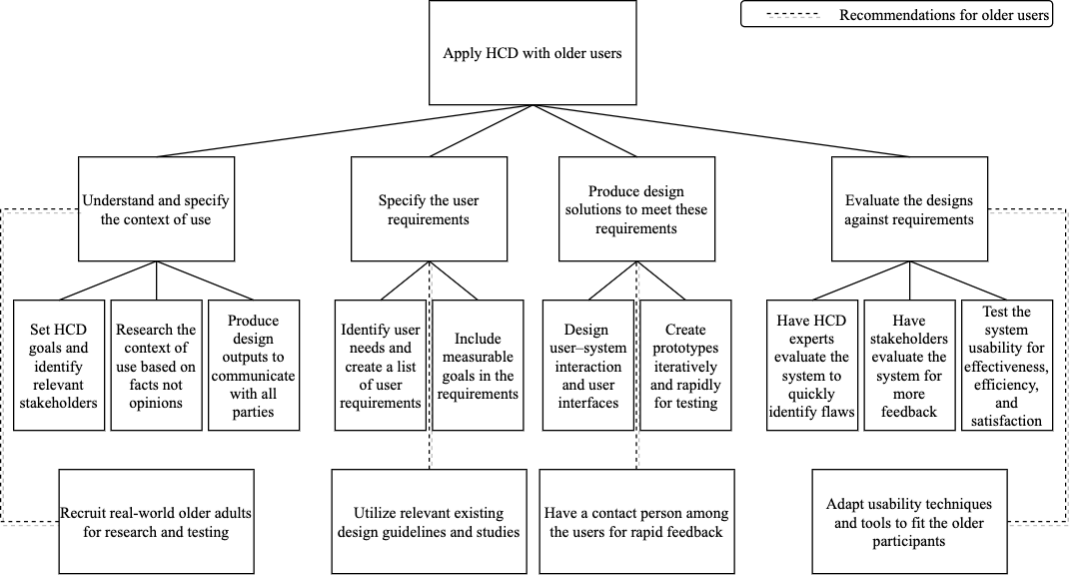
Pyramid model of HCD process with recommendations for older users by time order. HCD: human-centered design.

This systematic review has shown how HCD can be used to create mHealth for older adults, with additional recommendations reported. Eight studies are included in this review: 5 are mixed methods studies and 3 are case studies. All studies were published recently starting from 2017 onward, suggesting that the subfield is relatively new. All were conducted in developed countries and mostly in academic or specialized health care settings. Because of the diverse methodologies and details of the included studies, we used the Minto pyramid principle and the 4 HCD steps from ISO 9241-210 to guide the creation of 3 conceptual models: Figure 2 shows a structure of HCD team members and stakeholders in the HCD process, and Figure 3 shows how HCD can be applied to create mHealth solutions for older users. The following discussion explains the models further and also explores limitations with recommendations for future research.

First, mHealth ideas, either novel or of existing concepts, should be based on what the users need, not what the creators want. As illustrated in the included studies, the authors, usually acting as the management team that oversees the project, identify and base their proposed mHealth solutions on real stakeholders both directly [30-36] and indirectly [8]. The first step of HCD investigates whether the solution fits well with the target users; this step also aims to produce outputs that ensure all HCD team members and stakeholders share the same vision. Stara et al [36] conducted focus groups to discuss their preliminary concept with relevant stakeholders. Researchers, or the functional team members, then recruit relevant stakeholders, both health care providers and patients, to gain more insight into their context of use: the users, their environment, and their current activities.

The included studies’ details and rationales for the number of participants and the sampling method were diverse and vague. For example, Fortuna et al [8] had no participant and relied solely on a literature review to identify older adults’ needs, whereas Srinivas et al [35] had a total of 100 participants and remarked that the gathered data proved to be more than they used for design. However, it should be noted that the 2 studies differ in their design goals, where the first wanted to implement a known intervention on mobile phones (app), while the second sought to identify new problems from an existing routine entirely.

Besides quantity, most included studies recommended that the sampling method include diverse groups of participants to ensure HCD solutions reflect real-world problems. Reaching out to older adults who are more physically inept or socially disadvantaged can be challenging. Fortuna et al [8] tackled this by building on the results of existing research in middle-aged and older adults with serious mental illness. Cornet et al [30] suggested recruiting key stakeholders who know how to approach such a group of patients to help. Harte et al [32] used purposive sampling and evaluated their participants’ visual perception and cognitive processing to ensure the process was inclusive. Thus, the recruitment of older adults for HCD projects should be flexible and inclusive to best serve HCD goals.

Information on the context of use was mostly gathered through qualitative methods in the included studies. Observation is valued more than opinions in HCD: it shows how users currently pursue their goals from an unbiased perspective. Interviewing techniques that can be employed are (1) the critical incident technique, and (2) the think-aloud method of a fictitious scenario [30]. This factual information of the context of use is crucial to HCD as the functional team needs it to create HCD outputs such as personas and use-case scenarios to communicate with the technical team to develop a suitable mHealth solution. All 3 actions, which are (1) the management team setting design goals and identifying stakeholders, (2) the functional team gaining insight into the users, and (3) the functional team creating design outputs, can and should be done iteratively to truly understand the context of use.

Second, mHealth solutions need to address the current pain points of the users and ensure they achieve their intended goals; a clear understanding of user needs and a concise list of user requirements help the HCD team accomplish that. The context of use plays a vital role in identifying the user problems from their current activities and what the users need to solve them. Then, user requirements based on these user needs are created to guide the HCD team on how the solutions should be designed. These requirements can be obtained through a literature review [8] or a direct contextual inquiry of recruited participants [30,35]. For example, Srinivas et al [35] successfully derived user requirements from the user needs identified through thematic analysis of the established context of use.

The included studies also pointed out that there was a set of requirements unique to older users; however, most had not listed these requirements at the beginning and dealt with them only after the users raised the problems in usability testing. These design considerations for older adults are well-established: Harte et al [51] reported an extensive list of HCD considerations for connected health devices for older adults, and Li et al [52] identified barriers to mHealth adoption by older adults in their narrative review. With these guidelines, mHealth solutions can be designed to suit older adults’ physical and cognitive limitations prior to testing for efficiency. Psychosocial factors such as motivation, technology perception, and social influence need to be addressed as well to ensure adoption.

Specifying user requirements also means setting measurable goals for the mHealth system. This usually requires gathering quantitative data for the context of use, such as the duration to complete the conventional I-IMR, which is approximately 8-10 months [8]; the HCD team might set the goals for their system to take only 4-5 months accordingly. If no goal is set, the way to assess the system in the subsequent steps will be limited.

Third, HCD seeks to create an ideal system through iterative prototyping together with the stakeholders, to make certain all user needs and requirements are accounted for. In the beginning, the functional team should design how the users will interact with the system and how the interfaces will be like. Harte et al [33] demonstrated this in their study by creating use cases as outputs to be analyzed by stakeholders for feedback. These outputs are called low-fidelity prototypes because they are easy to create, simple to change, and able to quickly convey the design concepts to all relevant parties. Besides, user interfaces can be based on existing design guidelines, such as the literature review about the unique user needs and requirements of older adults done by Fortuna et al [8]. Once the design is refined and approved, the technical team could then create an interactive software of the system or high-fidelity prototypes.

It should be noted that high-fidelity prototypes are not open for major changes or costly to do so; the best approach would be to finalize user–system interaction and user interfaces before their creation. As this HCD step requires iteration, an agile approach is recommended [35]. Agile is a software methodology based on rapid and iterative prototyping to gain continuous feedback from users, allowing developers to quickly create, evaluate, and improve their solutions to best fit the users [53]. Communication with stakeholders is key in this step, especially with the older population whose participation tends to be low due to their technology ineptness, physical and cognitive limitations, and logistical issues, for example, timing and travel [30]. Cornet et al [30] recruited patient advisors, older adult volunteers, to bridge these gaps: these patient advisors were able to give rapid feedback from users’ perspectives. Due to HCD being agile in nature, this step is often done together with the evaluation step.

Fourth, evaluation comes after the production of design solutions. The functional team should work closely with the technical team to evaluate the produced solutions. In HCD, evaluating the designs is done by usability experts and end users. A number of included studies recommended system evaluation by usability experts before end users. Expert evaluation can identify and classify usability problems early in the process where changes are less punishing; it is also much simpler to arrange compared with its user-based counterpart [32,33,35]. The process can be conducted according to standards such as usability heuristics [30,32,33,35] or by having the experts role-play as real end users [32,33]. However, it should be noted that the greater the difference in usability knowledge between the experts and the users, the more divergent the results from the 2 groups might be [26].

Evaluation by end users is critical to the process because getting feedback from the target users and improving accordingly would surely make the system usable for them. This result can be obtained by recruiting the right participants. Fortuna et al [8] reported that their sampling method with the intention to select only willing participants to test the system could have excluded the group of real end users who might be less eager to participate. Harte et al [32] dealt with this problem by verifying that the recruited participants fit well with HCD goals: the participants’ visual perception and cognitive processing were measured to confirm that the sampling method was inclusive enough. As for the number of participants, no standard has been agreed upon, but 5-10 participants are typically enough to discover major usability problems [45].

User-based evaluation methods range from giving the users specific tasks in a controlled environment to letting them use the system in the real world; the complexity also increased respectively so. Cornet et al [30] remarked in their study that usability testing in a controlled laboratory setting is more prevalent in research as it is less complicated to set up; however, it also has limitations as the set time and place cannot replace the real intended context of use. Srinivas et al [35] discussed further in their study about the 2 laboratory-based methods: the task-based test is good for the identification of user interface flaws due to its straightforwardness in giving users a set of smaller tasks to complete, while the scenario-based test can help explore how the users perceive the system and its purpose in a similar way of using it in the real world. To summarize, laboratory-based usability testing is recommended during iterative prototyping, and researchers should then plan for usability testing in the real context of use if possible [30].

The included studies concurred that both qualitative and quantitative data should be interpreted together for robust usability evaluation results [8,30-36]. Qualitative data are gathered from interviews and participants’ statements during the process. These statements can be encouraged by utilizing usability techniques such as the think-aloud and verbal prompting methods. Quantitative data are collected through standardized usability tools and usability metrics. However, the older population might show resistance toward these usability techniques and tools [35]. Orientation sessions about the goals of these techniques prior to testing could ease the older users’ doubts [30], and adaptations of standardized tools such as using simpler synonyms and combining multiple tools into a single questionnaire could also help [8,30]. In addition to traditional means, Petersen et al [34] showcased the use of sentiment analysis and natural language processing to help analyze qualitative data in HCD; using such technology could improve the overall process, and more research is suggested.

ISO 9241-210 defines the components of usability as effectiveness, efficiency, and satisfaction [26]. Evidence on how HCD improves the usability of mHealth for older adults is still lacking as most included studies only report satisfaction based on SUS, with only 2 studies by Fortuna et al [8] and Harte et al [32] reporting objective usability metrics that represent effectiveness and efficiency of the system [8]. Setting baseline goals during the second step of specifying user requirements might help researchers draw more substantial conclusions.

Although limited, the positive outcomes from the studies in this review show that HCD can create usable mHealth systems for older adults. Stara et al [54] further suggested that this point held true even when the system was used in other cultural settings adjacent to the one it was developed in: the WIISEL system, which was developed in Ireland, also had good usability scores when tested in Israel and Italy. They added that these results meant the system’s usability demands were within the capabilities of the users in the 3 countries. Human capabilities can be divided into 4 categories: physical, sensory, emotional, and intellectual [55]. The older population shares limitations in all these aspects, and by carefully addressing their needs with HCD, designers can create universally accepted products for older users across the globe [51].

The author again emphasize that user involvement in HCD is paramount to obtain such outcomes. Older adults are not extra design challenges to solve. Empathy toward users as individuals with pain points is essential to HCD; stereotypes and bias against older people could lead to design failures if left unchecked [56]. To avoid such pitfalls, we have to learn from the *untold stories* [30]. This review has gathered and summarized practical HCD challenges and strategies from primary research to aid HCD implementation with older adults.

Hastened by the COVID-19 pandemic, the field of mHealth will only expand. Moving forward, digital health solutions are aiming further than empowering patients and enhancing delivery. They are going for “digital therapeutics.” These evidence-based interventions aim to prevent and manage medical conditions through digital platforms and mobile devices; one of its focuses is to deliver lifestyle therapy to combat chronic diseases such as type 2 diabetes [57]. Older adults are major target users as most have chronic conditions and can benefit greatly from these digital lifestyle therapies. However, the field is in need of solutions for effective development, testing, and deployment [58]. Future research on implementing HCD in digital therapeutics might be able to solve these issues and improve the health of the older population as a whole.

### Limitations

Limitations of this systematic review are acknowledged. First, the ACM Digital Library was not included in this review despite being in the relevant field. We did search the database on the same day as the others: no studies from the ACM Digital Library passed our criteria. We then failed to mention this once we proceeded with the review. However, we ran another search with the same strategy on the database in May 2021 to recheck; 14 studies found did not pass our abstract screening according to our established eligibility criteria.

Second, the research question aims to address the whole HCD process, but an existing body of literature proves to be limited as the topic is an emergent subfield, especially with older adults as the target group. Although the criteria are forgiving, the search strategy and the inclusion criteria still demand that all steps of HCD are implemented in each app development. This excludes a large number of studies that feature only a part of HCD. For example, one study might focus on qualitative interviews without applying them, while another might test a newly developed system that is based solely on the authors’ vision, not actual user needs. Nevertheless, the included studies complement one another and thus can accommodate the research question as illustrated in this review.

Third, the highly diverse HCD goals and methodologies in the included mHealth apps restrict the means of analysis and synthesis of results. All studies relied heavily on various qualitative means for HCD such as literature reviews, interviews, and field notes from direct observations. Even the seemingly same approaches, such as interviews, still differ in detail such as the time, the duration, the focus, and the questions. Most studies also focus more on the process not the result, or in the case of case studies, the process itself is the result. This might be due to the fact that baselines of the existing activities are not established in the second step of HCD, specifying the user requirements, so comparisons for effectivity and efficiency of the newly developed mHealth interventions cannot be made with objective metrics. Because of that fact, the included studies have to be reviewed with qualitative techniques using narrative synthesis and guided by ISO 9241-210 together with logical ordering of the Minto pyramid principle [26,37,38]. Quantitative results of the included studies, which are based on the less tangible satisfaction results of standardized tools and often lack a definite conclusion, are also underutilized.

This leads to the fourth limitation regarding the included studies: all but 1 of the 5 mixed methods studies are rated to be of inadequate quality by the MMAT. Their quantitative components lack clarity. They do not explain their sampling methods or have done so insufficiently, resulting in the inability to deem their samples representative of the target population and failure to address possible confounding factors in making the conclusion that HCD helps make a usable product. This issue of the sampling methodology is also raised by the authors of the included studies; future HCD research should note this point in their strategic planning accordingly.

Finally, the authors stress that the aim of this systematic review was not to assess the implementation of HCD in creating mHealth for older adults or the effectiveness of mHealth interventions. The objective was to explore existing literature and establish recommendations and pitfalls for subsequent HCD projects. The older adults might be a narrow target population, but being the more sensitive and vulnerable group, the insight gained could be applicable to a wider range of users and help make future mHealth solutions more inclusive as well.

### Conclusions

This review concludes that HCD can be used to create mHealth solutions for older adults and has summarized the process based on the 4 HCD steps with additional recommendations. The findings of this review can help designers, developers, and researchers gain an overview of HCD for older adults and implement the framework in their projects. The growing body of literature is encouraging, but more evidence-based results of HCD on creating mHealth for older adults are still needed. Future research should also focus on applying artificial intelligence and machine learning in HCD and utilizing the framework to create novel mHealth solutions for the population.

## Appendix

Multimedia Appendix 1 Preferred Reporting Items for Systematic Reviews and Meta-Analyses 2009 checklist of this systematic review.

## Abbreviations

ASQ: After Scenario Questionnaire
CIED: cardiac implantable electronic devices
HCD: human-centered design
HIPAA: Health Insurance Portability and Accountability Act of 1996
I-IMR: Integrated Illness Management and Recovery
ISO: International Organization for Standardization
mHealth: mobile health
MMAT: Mixed Methods Appraisal Tool
NASA-TLX: NASA-Task Load Index
NVS: Newest Vital Sign
PRISMA: Preferred Reporting Items for Systematic Reviews and Meta-Analyses
PSSUQ: Post-Study System Usability Questionnaire
QUEST: the Quebec User Evaluation of Satisfaction with Assistive Technology questionnaire
SUS: System Usability Scale
USE: Usefulness, Satisfaction, and Ease of use
WIISEL: Wireless Insole for Independent and Safe Elderly Living
